# Peptized Milk as Food for Infants and Invalids

**Published:** 1880-12

**Authors:** 


### PEPTIZED MILK AS FOOD FOR INFANTS AND INVALIDS.

NUNN recommends the following modes of preparing this valuable food: Take one pint of milk at 80° Fahr.; add a tea-spoonful of rennet solution or ten grains of pepsin, and keep the mixture at 80°. When coagulation is complete, but before the whey has begun to separate, beat the whole up smooth with a whisk or beater, and pass through a fine milk-strainer to insure the minute division of the curd. This preparation appears to keep equally as well or better than raw milk, remaining apparently unchanged for twenty-four hours if kept cool. Dilute and sweeten for feeding as usual.

By this method coagulation is complete, and no further change of that nature is requisite, the weakened stomach of the invalid receives the necessary nutriment carrying with it its own digestive principle.

